# Review of Intra-Arterial Therapies for Colorectal Cancer Liver Metastasis

**DOI:** 10.3390/cancers13061371

**Published:** 2021-03-18

**Authors:** Justin Kwan, Uei Pua

**Affiliations:** Department of Vascular and Interventional Radiology, Tan Tock Seng Hospital, Singapore 388403, Singapore; pua_uei@ttsh.com.sg

**Keywords:** colorectal liver metastasis, liver-directed intra-arterial therapy, hepatic arterial infusion therapy, trans-arterial chemoembolization, radioembolization

## Abstract

**Simple Summary:**

Colorectal cancer liver metastasis occurs in more than 50% of patients with colorectal cancer and is thought to be the most common cause of death from this cancer. The mainstay of treatment for inoperable liver metastasis has been combination systemic chemotherapy with or without the addition of biological targeted therapy with a goal for disease downstaging, for potential curative resection, or more frequently, for disease control. For patients with dominant liver metastatic disease or limited extrahepatic disease, liver-directed intra-arterial therapies including hepatic arterial chemotherapy infusion, chemoembolization and radioembolization are alternative treatment strategies that have shown promising results, most commonly in the salvage setting in patients with chemo-refractory disease. In recent years, their role in the first-line setting in conjunction with concurrent systemic chemotherapy has also been explored. This review aims to provide an update on the current evidence regarding liver-directed intra-arterial treatment strategies and to discuss potential trends for the future.

**Abstract:**

The liver is frequently the most common site of metastasis in patients with colorectal cancer, occurring in more than 50% of patients. While surgical resection remains the only potential curative option, it is only eligible in 15–20% of patients at presentation. In the past two decades, major advances in modern chemotherapy and personalized biological agents have improved overall survival in patients with unresectable liver metastasis. For patients with dominant liver metastatic disease or limited extrahepatic disease, liver-directed intra-arterial therapies such as hepatic arterial chemotherapy infusion, chemoembolization and radioembolization are treatment strategies which are increasingly being considered to improve local tumor response and to reduce systemic side effects. Currently, these therapies are mostly used in the salvage setting in patients with chemo-refractory disease. However, their use in the first-line setting in conjunction with systemic chemotherapy as well as to a lesser degree, in a neoadjuvant setting, for downstaging to resection have also been investigated. Furthermore, some clinicians have considered these therapies as a temporizing tool for local disease control in patients undergoing a chemotherapy ‘holiday’ or acting as a bridge in patients between different lines of systemic treatment. This review aims to provide an update on the current evidence regarding liver-directed intra-arterial treatment strategies and to discuss potential trends for the future.

## 1. Introduction

Colorectal cancer (CRC) is the fourth most common cancer amongst men and women in the United States. In 2019, there were an estimated 145,600 new CRC cases in the US, with 51,020 deaths resulting from the disease [1]. Despite excellent advances in the field through a combination of early detection from screening, lifestyle prevention and improved systemic and loco-regional treatment strategies, approximately 20% of patients will have colorectal liver metastasis (CRLM) at first presentation [1], and more than 50% of patients will eventually develop CRLM during the course of their disease [2]. Notably, the liver is the most common site for metastasis from CRC, occurring in more than 70% of patients with metastatic disease, and is the most frequent cause of death related to this cancer [3]. Surgical resection of CRLM is the only potentially curative option and is considered whenever possible, demonstrating overall 5-year survival of 25–40% [4] compared to patients who are either untreated or undergo systemic chemotherapy alone, whom rarely survive past 5 years [5]. Unfortunately, only 20% of patients at presentation are considered to be potential surgical candidates for CRLM resection [6,7]. Further, patients who undergo a successful hepatic metastectomy have a fair probability of developing intrahepatic metastasis recurrence (approximately 30%), with a significant proportion of the recurrences developing within the first 2 years after resection [8].

For the majority of patients with unresectable CRLM, the mainstay of treatment is a doublet or triplet combination of systemic chemotherapy comprising of fluropyrimides (e.g., 5-Flurouracil (5-FU)), leucovorin and/or either oxaliplatin (FOLFOX) or irinotecan (FOLFIRI), with or without targeted therapies (e.g., anti-epidermal growth factor receptor (EGFR) for Kirsten rat sarcoma (KRAS) wild-type tumors and anti-vascular endothelial growth factor (VEGF) agents). These combination treatments have been associated with a significant improvement in overall survival (OS), reaching a median duration of over 30 months in certain studies [9]. Triplet chemotherapy regimens have been investigated (FOLFOXFIRI) with improved median progression-free survival (PFS) times and response rates (RR) at the expense of increased reports of systemic toxicity [10].

Over the past few decades, the field of interventional oncology has established its position as one of the four pillars of cancer patient care alongside surgical, medical and radiation oncology, offering clinicians an ever-growing armamentarium of local and loco-regional therapies to improve local disease control, prolong overall survival and palliate symptoms. This especially holds true in patients who progress despite systemic therapy. Liver-directed intra-arterial therapies (IAT), such as hepatic arterial infusion chemotherapy (HAIC), trans-arterial chemoembolization (TACE) and selective internal radiation therapy (SIRT), are inherently attractive options for patients with liver-only or liver-dominant metastatic disease due to the dual blood supply (70% portal and 30% arterial) to the liver parenchyma and the almost exclusive hepatic arterial supply to CRLM [11,12]. Thus, this allows preferential delivery of therapeutic agents directly to the tumor whilst minimizing significant hepatic and systemic toxicity. IATs have traditionally been considered in the salvage setting in patients with chemo-refractory disease, although in recent years, much research has investigated their use both in an adjuvant setting (for HAIC) as well as in a first-line setting in conjunction with systemic chemotherapy.

Current National Comprehensive Cancer Network (NCCN) and European Society of Medical Oncology (ESMO) guidelines recommend IATs in highly selected chemo-refractory patients with predominant liver metastatic disease [13,14]. ESMO guidelines also further stipulate that there may be a possible role for TACE or SIRT in a neoadjuvant and adjuvant setting, although this particular application should be limited to clinical trials at present. A note is made that in the case of SIRT, the available evidence leading to its inclusion in these guidelines are modest at best, with none of the randomized studies conferring a significant OS advantage when compared to the current standard of care [15,16]. Interestingly, Yytirum-90 (Y-90) SIRT has also garnered evidence in the latest NCCN guidelines (2A recommendation) to support contralateral liver lobe hypertrophy in patients who are being considered for resection with insufficient remnant liver volume [17]. This recent change is a clear indication of the potential growth for the role of IAT, not only limited to patients with unresectable disease. As there is an ever-growing body of evidence from the literature, their role in this treatment paradigm remains to be defined, especially with much recent emphasis placed on tumor biology, both for prognostication purposes and improving treatment outcomes. The focus of this review is to provide an update on the current evidence on each of the treatment modalities as well as to discuss potential trends for the future.

## 2. Hepatic Arterial Infusion Chemotherapy (HAIC)

HAIC has been available since the late 1980s and has been extensively studied in patients with CRLM. As the name implies, chemotherapeutic agents are infused directly into the hepatic artery via a surgically or percutaneously implanted catheter connected to a hepatic arterial port/external pump. This consequently allows preferential drug delivery directly to CRLM with relative sparing of the background liver parenchyma [12] and allows drugs to circumvent the first-pass effects of hepatic excretion, thus exposing tumor cells to a significantly higher concentration of chemotherapy as well as limiting systemic toxic effects. To put this in perspective, intra-tumoral concentrations of flurodeoxyuridine (FUDR), a 5-FU derivative, which is the most commonly used agent in the United States, can be up to 400-fold greater when compared to systemic infusion due to its high (95%) first-pass hepatic excretion [18]. Other agents such as oxaliplatin and irinotecan, already used in the first and second-line systemic treatment for unresectable CRLM, have also been trialed in HAIC, mostly in Europe and Asia, despite having a less favorable pharmacokinetic profile when compared to FUDR, and a different toxicity profile [19,20,21,22]. One fundamental principle of HAIC is that the hepatic artery has to be skeletonized such that the whole liver is supplied by a single artery. This usually requires surgical ligation (in the setting of surgical port placement) or permanent, proximal embolization (in the setting of percutaneous port placement) of the gastroduodenal artery (GDA) and the right gastric artery to prevent non-target mucosal injury, as well as toxicity due to extrahepatic drug perfusion [23,24]. The tip of the infusion catheter is usually parked in the gastroduodenal artery with a perfusion side hole in the hepatic artery proper to prevent dislodgement and to enable bilobar hepatic infusion [23]. If the catheter cannot be placed in the GDA, either for anatomical reasons or from previous ligation, the catheter is placed in a peripheral side branch of the hepatic artery. Whilst HAIC therapy has its appeals, initiation of therapy as well as administration can be complex. As such, it is likely that this treatment modality will only be offered at high-volume surgical oncology institutions with an established multidisciplinary infrastructure [25,26].

### 2.1. Evidence for HAIC in Unresectable CRLM

In patients with unresectable CRLM, the effectiveness of HAIC with combination systemic therapy has shown to improve local RR as well as OS. For this article, RR is based on the response evaluation criteria in solid tumors (RECIST) unless otherwise stated. The pertinent evidence on HAIC in this review is summarized in Table 1. In 2001, Kemeny et al. [27] conducted a Phase I study investigating HAIC with FUDR/dexamethasone with systemic irinotecan in 46 patients previously treated for unresectable CRLM (41% of the study cohort had previously been treated with two lines of systemic treatment) and showed a high RR of 74% (according to the World Health Organization (WHO) response criteria), with a median OS of 20 months. In a subsequent trial in 2005 [28], the same group conducted a separate phase I study in 36 patients, examining HAIC with FUDR/dexamethasone combined with systemic oxaliplatin and either irinotecan (21 patients) or fluorouracil/leucovorin (15 patients). Notably, in this cohort, almost 90% of patients were previously treated with systemic chemotherapy. For the irinotecan group, RR was observed in 90% (WHO criteria) of patients with median OS of 36 months, and for the fluorouracil/leucovorin group, RR was 87% with median OS of 22 months. Interestingly, 7 patients (19%) in the irinotecan group had a good enough response to be down-staged for liver resection, probably contributing to the longer OS in this group.

Promising data obtained from these early trials gave rise to further emphasis on the potential to convert initially unresectable CRLM to resectable/ablatable disease, essentially in a neoadjuvant setting. In a Phase I trial investigating conversion to resectability rates (CTR) [29], forty-nine patients with unresectable CRLM (53% previously treated with systemic chemotherapy) were treated with HAIC with FUDR/dexamethasone plus systemic oxaliplatin and irinotecan. Overall RR was 92% (WHO criteria) and CTR rate was 47% (57% in patients who were chemotherapy-naïve). On examining the patients that underwent resection, 73% patients had more than 5 lesions, 98% had bilobar disease and 65% had more than 50% of the liver involved—none of these variables reflecting extensive tumor burden were significantly associated with the probability of resection. The results of this study were further validated in a prospective phase II trial evaluating the long-term results and curative potential for patients treated with combination HAIC and systemic chemotherapy [34]. In this study of 64 patients (67% previously treated with systemic chemotherapy) with a median follow-up time of 81 months, thirty-three patients (52%) were converted to resection, doubling the historical rate, with a median PFS and OS of 13 and 38 months, respectively. Further, patients who underwent CRLM resection had a significantly longer 5-year OS of 63% compared to 13% in unresected patients. In a similar prospective study by Goere et al. [30] of 87 patients (79% previously treated with systemic chemotherapy) with unresectable CRLM treated with oxaliplatin HAIC and systemic 5-FU and leucovorin, the CTR rate was 26%. In patients who underwent resection, median OS of 41.7 months and 5-year OS of 56% were achieved. This study also demonstrated that CTR rates were significantly higher in patients who received HAIC in the first-line setting (53%, 10/19 patients) compared to those that received HAIC after failure of prior systemic chemotherapy (19%, 13/68 patients). In a European phase II multi-center trial (OPTILIV) [32], sixty-six patients (28 patients enrolled as second-line treatment and 36 patients enrolled as third/fourth-line treatment) were treated with a triplet combination of HAIC comprising of irinotecan, oxaliplatin and 5-FU with intravenous cetuximab. Patients had a median of 10 liver metastases involving a median of 6 segments. CTR was 30% with a median OS of 25.7 months in all patients. In a separate analysis on the OPTILIV trial [35], a RR of 63% was achieved in patients on second-line treatment and a RR of 38% and OS of 15.2 months in patients on third/fourth-line treatment.

The effectiveness of HAIC in heavily pre-treated patients is supported in several studies. In a retrospective review [33] of 110 patients with disease progression failing at least 3 prior standard systemic therapies, HAIC with concurrent systemic treatment showed a RR of 35% and an average OS of 16.3 months (20 months in patients with liver metastasis only and 11.4 months in patients with low-volume extrahepatic disease), which is significantly higher when compared to historical data in patients with chemo-refractory disease. In a separate study by Lévi et al. [31], fifty-six patients who had progressed on a median of three prior chemotherapy regimens were treated with chronomodulated HAIC with a combination of 5-FU, irinotecan and oxaliplatin with intravenous cetuximab, demonstrating an objective RR of 32% and a median OS of 13.7 months.

In short, there is compelling evidence from numerous studies that HAIC in combination with systemic chemotherapy is effective at achieving high local tumor RR with encouraging tumor downstaging potential and seems to be of benefit when applied earlier on in the course of disease. Large phase III trials are however currently lacking in this field and are required to validate these results, especially comparing HAIC with systemic chemotherapy vs. systemic chemotherapy alone. Further research is also ongoing into the prognostic effects of molecular markers (e.g., *RAS* and *BRAF*) on the outcome of HAIC as well as the addition of different targeted biological agents to HAIC regimens.

### 2.2. Evidence for HAIC in the Adjuvant Setting

In the adjuvant setting, early studies on the efficacy of HAIC yielded mixed results. A meta-analysis in 2006 [36] of 7 RCTs included 592 patients examining the efficacy of adjuvant HAIC conferred no significant advantage in OS between the pooled groups. However, in most of the studies, less than half of the patients were able to receive the complete planned HAIC protocol and trials also differed in the type of HAIC drug administered as well as the use of concurrent systemic chemotherapy. Despite this, the intrahepatic recurrence rates were doubled in the control group vs. the group receiving HAIC. In a 21-year analysis (1992–2012) of 2368 consecutive patients who had perioperative HAIC after curative-intent CRLM resection [37], median OS for patients with HAIC was 67 months vs. 44 months for those without. Overall, 5-year and 10-year OS for patients receiving HAIC compared to those without were 53% and 38% (*p* < 0.001) vs. 38% and 24% (*p* < 0.01), respectively. Despite this study spanning across the era prior to modern systemic chemotherapy, greater OS was still seen in the HAIC arm after propensity score matching. In a retrospective review of 98 patients [38] who had adjuvant oxaliplatin HAIC combined with systemic 5-FU vs. patients who received modern systemic chemotherapy alone, 3-year disease-free survival (DFS) was significantly longer in patients in the HAIC group compared to the systemic chemotherapy group (33% vs. 5%, *p* < 0.0001). Multivariate analysis confirmed that adjuvant HAI chemotherapy as well as R0 resection margin status were the only independent predictive factors for prolonged DFS. Whilst there was a trend for improved 3-year OS in the patients who received HAIC (75% vs. 62%, *p* = 0.17), this result was not statistically significant, possibly due to the patients in the HAIC group only receiving concurrent monotherapy. Currently, there are two RCTs underway to further evaluate the role of HAIC with systemic chemotherapy in the adjuvant setting. Postoperative Hepatic Arterial Chemotherapy in High-risk Patients as Adjuvant Treatment After Resection of Colorectal Liver Metastases (PACHA-01) is an ongoing phase II/III [39] trial in Europe with an estimated completion date in 2028, comparing adjuvant oxaliplatin HAIC with systemic 5-FU vs. systemic FOLFOX in patients with a high risk of CRLM recurrence (defined as having at least 4 prior resected CRLM). The primary endpoint is 18-month hepatic recurrence-free survival (RFS) rate with secondary objectives assessing feasibility, toxicity and efficacy. If hepatic recurrence RFS is successful in phase II, then the study will pursue to phase III, for which the primary endpoint will be 3-year RFS rate. The second study is the Adjuvant hepatic arterial infusion pump chemotherapy after resection of colorectal liver metastases in patients with a low clinical risk score (PUMP) trial [40], an open-label, phase III multi-center RCT in the Netherlands, evaluating the efficacy of adjuvant HAIC with systemic 5-FU compared to no systemic adjuvant therapy (standard of care in the Netherlands) in low-risk patients with resectable CRLM. The primary endpoint is PFS with secondary objectives of OS, hepatic PFS, safety and quality of life (QoL).

### 2.3. Complications and Toxicity Related to HAIC

Complications of HAIC can be divided into catheter-related complications and toxicity related to chemotherapy administration. Catheter-related complications include catheter migration, occlusion, hepatic arterial occlusion, extrahepatic perfusion or catheter/port-related infection, which is consistently reported in approximately 10–20% of patients in the literature. In a review of 544 consecutive patients from 1986 to 2001 with surgically implanted pumps [41], there was an overall catheter/pump-related complication rate of 22%. Notably, as the study period was over 25 years, complication rates significantly improved over the latter half of the study (1994–2001), probably due to increased operator experience as well as improved technique. As percutaneous techniques evolved, some authors sought to investigate whether catheter-related complications occurred less via a percutaneous implantation technique. Addressing this issue, a retrospective study of 126 patients was conducted in 2011 [42] comparing the complications related to percutaneous and surgically implanted catheters. While the investigators found that patients with percutaneous implanted catheters required an overall shorter hospital stay (1.8 days ± 0.7 vs. 8.2 days ± 22) and lower analgesic requirement (2.0 doses ± 0.9 vs. 9.7 doses ± 3.2), there was no significant difference in the number of catheter-related complications per chemotherapy course (percutaneous 9% vs. surgical 8%, *p* = 0.9) or rates of HAIC discontinuation (percutaneous 12% vs. surgical 19%, *p* = 0.12). In a more recent phase II multi-center study conducted in Japan [43], the efficacy and adverse events of HAIC using percutaneous catheter placement were evaluated. There was an overall catheter-related complication rate of 19%, of which HAIC treatment was discontinued due to catheter/procedural-related complications in 15.6% of patients (12/77 patients)—this was similar to the reported rates of surgically implanted catheters in previous studies. In a comprehensive review of 4580 cases in 2001 [44], the most common drug toxicities related to HAIC were gastrointestinal symptoms (22%), chemical hepatitis (19%) and bone marrow toxicity (8%). Hepatobiliary toxicity evident by serum transaminitis, hyperbilirubinemia and biliary sclerosis were also serious issues, with a higher incidence in FUDR [45,46]. To mitigate this risk, intra-arterial dexamethasone was administered together with FUDR. In a randomized study of 50 patients, those receiving dexamethasone plus FUDR had a trend towards decreased hyperbilirubinemia (*p* = 0.07) with increased response rate (*p* = 0.03) [47]. Alternatively, as biliary sclerosis is not seen with 5-FU HAIC, some investigators have toggled between regimens of FUDR HAIC and 5-FU HAIC to reduce hepatotoxic effects that may lead to early treatment termination [46,48].

## 3. Trans-Arterial Chemoembolization (TACE)

TACE is a trans-arterial, catheter-based locoregional treatment to the liver, combining injection of chemotherapy drug and embolic material directly into the hepatic artery. The rationale of TACE is two-fold; firstly, to deliver a high dose of chemotherapeutic drug directly to tumor tissue, thereby reducing systemic toxicity, and secondly to interrupt blood supply, causing ischemia and necrosis. These mechanisms are supposedly synergistic in nature, allowing delayed washout of the anti-tumoral agent and enabling a prolonged duration of action. TACE has long been established as a first-line treatment for Barcelona Clinic Liver Cancer (BCLC) intermediate stage hepatocellular carcinoma (HCC) [49], although in the setting of CRC, the primary indication for TACE is as second-line treatment in patients with chemo-refractory CRLM with liver-only metastatic disease.

In TACE, the technique and type of chemotherapeutic/embolic agent injected varies across different centers. Regardless, the principle remains the same: vascular access is first obtained either from the common femoral or radial artery. A catheter is passed through the celiac trunk into the main hepatic artery where standard, high-quality angiography is performed, prior to delivery of the chemo-embolic drugs. The angiogram serves several purposes: to delineate the hepatic arterial anatomy and potential variations, determination of tumor arterial feeders, identification of arteries to be avoided during treatment delivery (e.g., right gastric, gastroduodenal artery, to prevent inadvertent non-target embolization) and evaluation of portal vein patency [50]. Modern techniques include the use of cone-beam computed tomography (CBCT) to significantly increase detection of tumors as well as tumor feeding arteries during TACE [51]. After the chemo-embolic drugs are delivered, this is usually followed with an embolizing agent (e.g., Gelfoam, polyvinyl alcohol (PVA) or calibrated microspheres). The optimal endpoint of TACE has not been formally established, but generally, most interventionalists would embolize until stasis, or near stasis, achieving a ‘tree-in-winter’ appearance [52]. Multiple treatment sessions are often required, alternating in a lobar fashion, especially in the setting of bilobar disease to ensure satisfactory treatment, as well as to prevent drug toxicity and avoid potential hepatic decompensation related to the embolization.

The use of conventional TACE (cTACE) for unresectable CRLM, which is chemotherapeutic drugs mixed with Lipiodol (Guerbet, Paris, France) to form a liquid emulsion, was largely adopted from its use in HCC. Common drugs used in cTACE include Mitomycin, Cisplatin and Doxorubicin. With the advent of drug-eluting bead (DEB, DC/LC Bead, BTG, UK Ltd.) technology, the use of cTACE alone in the setting of CRLM dwindled, probably related to limited efficacy for increasing OS and the lack of prospective trials. DEB-TACE utilizes calibrated microspheres loaded with cytotoxics (e.g., irinotecan) to allow a more prolonged and reproducible manner of drug release [53]. The use of DEB also results in a lower peak plasma concentration of chemotherapeutic drugs compared to cTACE, essentially reducing systemic side effects and local toxic effect to healthy liver parenchyma [54]. In the context of CRLM, the results obtained with DEB loaded with irinotecan (DEBIRI) have been promising and will be discussed specifically in a later section. To date, there is no head to head comparison of DEBIRI-TACE to cTACE, thus, the decision to offer one over the other is often a matter of institutional preference [55].

### 3.1. Evidence for Bland Embolization (TAE)

The use of trans-arterial bland embolization (TAE) for unresectable CRLM has been investigated, although data on the subject is limited and to our knowledge is not widely used. A prospective study in 1990 evaluating 61 patients with unresectable CRLM, randomized to either bland-TAE (using gelatin sponge and autologous lyophilized dura matter), HAIC or no treatment showed no significant survival benefit in the TAE arm [56]. In a separate study evaluating the advantages of DEBIRI in a rat colorectal liver metastases model [57], embolization with DEB alone showed no effect on tumor burden, compared with a dose-dependent response with DEBIRI. Findings from this study suggest that both the effects of drug and embolization are important to achieve a tumoricidal response. In contrast, a pilot study by Tanaka et al. evaluated the response of repeated bland-TAE with 100 um calibrated microspheres administered through an implantable port-catheter system, in two heavily pre-treated patients with unresectable CRLM [58]. The procedure was repeated four times in intervals of 14–21 days due to the initial observation of hepatic arterial recanalization and recurrent tumor staining 13 days after the first TAE procedure. Two-month computed tomography (CT) follow-up after the procedures for both patients showed tumor necrosis and an OS approaching 6 months without any additional therapy. There may be an ongoing role for bland-TAE in combination with percutaneous ablation for oligometastatic, unresectable CRLM, where several studies have shown the synergistic effects of combining both modalities to achieve larger ablation volumes and reducing peripheral recurrence [59].

### 3.2. Evidence for cTACE

In the largest series to date evaluating the long-term results of cTACE in 564 patients with unresectable, chemo-refractory CRLM, overall RR was 65% with reported 1-, 2- and 3-year survival rates of 62%, 28% and 7%, respectively (median OS of 14 months) [60]. TACE was used during second-line treatment or later, and patients were treated for an average of 6 sessions (range: 3–29). Chemotherapy drug protocols included Mitomycin C, Mitomycin C/Irinotecan, Mitomycin C/Gemcitabine or a combination of Mitomycin C/Irinotecan/Cisplatin with embolization performed using Lipiodol and starch microspheres. There is currently no formal study evaluating the use of cTACE in the first-line setting. However, a study by Albert et al. [61], evaluating 121 patients with cTACE, included 63 patients (52%) who had undergone 0–1 line of prior systemic treatment using Cisplatin, Doxorubicin and Mitomycin C mixed with Lipiodol and PVA particles. Not surprisingly, patients who had 0–1 lines of prior systemic treatment fared better than those who had 3–5 lines of prior treatment, with a median OS of 12 months and 6 months respectively, with an overall RR of 43% and a median OS of 9 months after the start of treatment for all patients. Results from another study evaluating 66 patients (46% chemo-naïve) treated with 5-FU/GM-CSF/Melphalan with Lipiodol and Gelfoam reported an overall RR of 88% (WHO criteria) [62]. Median survival for this study, however, was not reached, as only 66% of patients survived at 2 years. Overall, the available data on cTACE shows limited efficacy at increasing OS, especially in the era of modern systemic chemotherapy. Together with the lack of prospective trials, the use of DEBIRI-TACE seems to have superseded cTACE alone in the setting of unresectable CRLM. There does however appear to be a role for cTACE in conjunction with other therapies, such as percutaneous ablation for the treatment of oligometastatic, unresectable CRLM. In a retrospective review of 452 patients undergoing repetitive cTACE, two-hundred and nineteen patients were treated in a neoadjuvant setting followed by percutaneous thermal ablation [63]. This group of patients fared better in terms of median PFS and OS compared to those treated with cTACE only in a palliative setting (11 months and 26 months vs. 6 months and 13 months, respectively). In a separate phase II study examining 25 patients treated with TACE (degradable starch microsphere and Mitomycin C) followed by radiofrequency ablation (RFA), 2-year local tumor control rate was 92%, with a median OS of 48.4 months [64].

### 3.3. Evidence for DEBIRI-TACE

The data on the efficacy of DEBIRI TACE is more established based on a series of limited Phase II trials [65,66,67,68], as well as a randomized phase III trial [69], summarized in Table 2. In a multicenter, single-arm prospective study of 55 patients treated with multiple prior lines of chemotherapy who underwent a total of 99 DEBIRI treatments (median of 2 sessions, range: 1–5), overall RR was 75% at 1 year, with PFS and OS of 11 months and 19 months, respectively [65]. In a separate study, Aliberti et al. evaluated 82 heavily pre-treated patients (failed at least 2 lines of prior systemic chemotherapy) treated with DEBIRI. Median follow-up time was 29 months with reported RR of 78% at 3 months, PFS and OS of 8 months and 25 months, respectively [66]. In a more recent prospective phase II study also evaluating heavily pre-treated patients, Izzei et al. [67] evaluated 20 patients who had progressed on at least 2 prior standard lines of chemotherapy with an additional 50% of patients having failed 3 or more lines. An objective RR of 60% (according to modified RECIST 1.1 (mRECIST)) was reported with a median PFS and OS of 4 months and 7.3 months, respectively. The less favorable outcome achieved in this study could have been related to several factors, such as the small sample size as well as 40% (8 out of 20 patients) of patients discontinuing treatment before completion. The efficacy of DEBIRI in addition to a biological agent alone, such as bevacizumab, has also been examined. In a prospective study of 30 pre-treated patients [68], randomized to either DEBIRI alone or DEBIRI with bevacizumab, the addition of bevacizumab showed an increase in RR (77% vs. 19%, *p* < 0.01), PFS (6 months vs. 4 months, *p* < 0.01) and median OS (12 months vs. 5.8 months, *p* < 0.01) when compared to the DEBIRI alone arm. In the only small phase III RCT conducted by Fiorentini et al. [69], seventy-four patients with liver-only metastatic CRLM were randomized to receiving DEBIRI or FOLFIRI. All patients enrolled had undergone at least 2 lines of prior systemic chemotherapy. Patients undergoing DEBIRI had two treatment sessions for each involved lobe, one month apart. Whilst some have questioned the statistical rigor of this study, there was an overall increase in OS of 7 months in patients treated with DEBIRI vs. FOLFIRI (22 months vs. 15 months respectively, *p* = 0.031), as well as improved PFS in the DEBIRI arm (7 vs. 4 months, *p* = 0.006). On examining QoL, patients treated with DEBIRI also enjoyed longer duration QoL with less significant side effects (8 vs. 3 months, *p* < 0.001).

In the first-line setting, Martins et al. evaluated 10 chemo-naïve patients in a phase I study treated with DEBIRI and systemic mFOLFOX ± bevacizumab [72]. Despite a small sample size, the results were impressive with a 12-month RR of 100% and median OS of 15 months: four patients (40%) were down-staged successfully to undergo secondary hepatic CRLM resection with an overall pathological response rate of 90–95% [73]. This high degree of pathological necrosis was mirrored in the Chemoembolization Using Irinotecan Bead Prior to Surgery in Metastatic Colorectal Cancer (PARAGON-II) study [74], where 40 patients with easily resectable CRLM were treated with a single session of DEBIRI, 1 month prior to surgery in a neoadjuvant setting. Seventy-six percent of targeted, resected lesions showed a histologically major or complete pathological response. In a randomized phase II trial of 70 patients evaluating DEBIRI in combination with systemic mFOLFOX ± bevacizumab as compared to FOLFOX ± bevacizumab alone in chemo-naïve patients, there was an overall improvement in 6-month RR in the DEBIRI arm (76% vs. 60%, *p* = 0.05), which led to a significantly higher CTR rate (35% vs. 16%, *p* = 0.05) and longer median PFS (15 vs. 8 months, *p* = 0.18), albeit non-significant [70]. No significant difference in chemotherapy-associated adverse events were reported between the study arms. A more recent phase II study [71] evaluated 57 chemo-naïve patients with liver-dominant CRLM who received both DEBIRI and mFOLFOX6. The majority of patients received 4 DEBIRI sessions, in an alternating fashion between the right and left lobe, although 37% patients had bilobar treatment in 2 sessions which subsequently resulted in more serious toxicities encountered in this subgroup. Despite the study not meeting its endpoint of >75% PFS at 9 months (actual 9-month PFS of 54%), objective RR of 73% and median PFS and OS of 10.8 months and 37.4 months respectively, were demonstrated. Notably, nineteen patients (33%) were suitably down-staged to undergo a secondary R0 resection with or without ablative therapy.

### 3.4. Complications of TACE

Approximately 30% of patients undergoing DEBIRI-TACE develop mild adverse complications, with the most common being abdominal pain, hypertension, nausea and vomiting [75]. Post-embolization syndrome can occur in up to two thirds of patients, characterized by abdominal pain, nausea, fatigue, transient derangement in liver function and fever without sepsis in the peri- or post-treatment period [76]. Severe complications such as hepatic abscess, hepatic failure, pancreatitis and peptic ulceration are rare with gastrointestinal ischemia related to non-target embolization, occurring in <1% of patients [23]. Bile duct injury has been reported in up to 11% of patients undergoing TACE, due to the bile ducts being solely perfused by the hepatic arterial vasculature [77]. Due to the local delivery of chemotherapy, systemic drug toxicity is rare. An increase in adverse complications and prolonged hospital stay have been associated with the lack of hepatic arterial lidocaine use, three or more treatments, obtaining complete stasis during TACE, dose of >100 mg of DEBIRI per treatment session, serum bilirubin > 2.0 mg/dL and >50% tumor involvement of the liver [78]. When comparing DEB-TACE to cTACE, both have a similar toxicity profile, although DEB-TACE has demonstrated fewer drug-related adverse events when compared to cTACE [79].

## 4. Y-90 Selective Internal Radiation Therapy (SIRT)

The role for external beam radiation therapy in the treatment of primary and secondary liver tumors has historically been limited by the low-radiation threshold of normal, healthy liver parenchyma [80]. Given this limitation, and the need for significantly higher radiation doses to achieve a tumoricidal effect, SIRT has emerged over the past few decades as a treatment option for patients with unresectable liver tumors. Micron-sized spheres (20–60 μm) loaded with a radioisotope are introduced using percutaneous trans-arterial techniques directly into the hepatic arterial vasculature supplying tumor tissue [81]. Yytrium-90 (Y-90), a pure beta-emitter, is the most commonly used radionuclide to label microspheres due to its favorable characteristics: mean tissue penetration of 2.5 mm (maximum 11 mm) and relatively short half-life of 64.2 h, thus allowing delivery of high-radiation doses (as high as 150 Gy) to tumor tissue, whilst limiting the radiation effects to the background liver parenchyma [82]. There are currently two commercially available microspheres composed of either glass (Therapshere^®^; BTG International, London, UK) or resin (SIR-Spheres^®^; Sirtex Medical, North Sydney, Australia). SIR-Spheres gained full premarketing approval by the Food and Drug Administration (FDA) in 2002, for the treatment of unresectable CRLM in conjunction with intrahepatic FUDR. This was largely based on the results of a randomized phase III trial in which 74 patients with liver only, unresectable CRLM were assigned to receiving either HAI-FUDR alone or HAI-FUDR with a single SIRT administration [15]. Patients in the combination SIRT arm demonstrated significantly longer median time to progression (TTP) (15.9 months vs. 9.7 months in the HAI-FUDR alone arm) as well as demonstrated higher OS rate at 1, 2 and 3 years (72%, 39% and 17% vs. 68%, 29% and 6.5%), albeit not statistically significant. In contrast, Therapsheres gained approval by the FDA in 1999 under a humanitarian device exemption for the treatment of unresectable HCC in patients with appropriately positioned hepatic arterial catheters [81]. In a practical sense, there are physical differences between the two types of spheres, mainly size, and potential activity per sphere which affects the embolic potential as well as dosimetry for treatment planning. In the setting of CRC, Y-90 SIRT has primarily been used in patients with liver-only or liver-dominant unresectable CRLM, with chemo-refractory disease. However, in recent years, its role as first-line treatment in combination with systemic chemotherapy, as well as in a neoadjuvant setting in patients with initially unresectable CRLM, have also been examined.

Technically, Y-90 SIRT treatment is divided into two parts: mapping and treatment. This is usually performed up to 2 weeks apart through two outpatient visits, or in some centers, all performed on the same day [83]. For the mapping stage, once vascular access is obtained, a hepatic angiogram is performed to assess the hepatic vasculature and identify the vessels supplying the tumors. In some cases, this may also include identification of any parasitized tumor-supplying vessels, arising from extra-hepatic arteries. In this context, these are usually coil-embolized during this stage, to consolidate tumoral blood supply via the main intrahepatic arteries [84]. It is also of critical importance to identify arteries supplying extrahepatic structures such as the stomach, duodenum and pancreas to avoid non-target deposition of Y-90 radioactive microspheres during the subsequent treatment session, which could result in serious complications such as non-healing ulcers and pancreatitis [85]—these vessels are either avoided, or prophylactically embolized at this stage. Once the desired microcatheter position(s) is chosen, technetium-99 (^99^mTc)-labelled macroaggregated albumin (MAA) is injected as a surrogate, into each artery planned for Y-90 microspheres delivery. The purpose of this is two-fold, first, to calculate lung shunt fraction (LSF) using planar scintigraphy, and second, to perform a single photon emission CT (SPECT) image to evaluate for inadvertent gastrointestinal deposition as well as to predict intrahepatic tumoral microsphere distribution [55]. Patients with LSF of more than 20%, corresponding to greater than 30 Gy of radiation delivered to the lungs, cannot undergo SIRT due to the potential risk of irreversible radiation-induced pneumonitis. During the treatment session, the Y-90 microspheres are administered at the planned microcatheter position(s), and thereafter, a bremsstrahlung single photon emission computed tomography (SPECT) study is performed to confirm satisfactory tumor coverage [86]. In the majority of patients who have bilobar disease, whole liver treatment can be performed in a single session, or in two sequential lobar treatments 4–8 weeks apart if the patient’s liver reserve is in question. In practice, single-session treatments are preferred for logistical reasons, as well as to prevent the risk of non-target Y-90 microsphere deposition due to potential interval change in the hepatic arterial vasculature between treatment sessions.

### 4.1. Evidence for Y-90 SIRT in Chemo-Refractory Patients

The relevant data on Y-90 SIRT is illustrated in Table 3, with the majority of data pertaining to patients who have chemo-refractory disease. Y-90 SIRT has shown the most benefit in this subset of patients, demonstrating objective RR of 10–48%, based on RECIST criteria, and an OS ranging from 9.6 to 14.9 months [16,87,88,89,90,91,92,93,94,95]. This is encouraging, as the median OS in chemo-refractory patients is approximately 4–6 months with current systemic treatment options such as TAS-102 [96] and Regorafenib [97], which have shown a modest but significant OS benefit in phase III trials of approximately 2 months when compared to best supportive care. In a phase I dose-escalation study of 25 patients’ refractory to systemic first-line 5-FU, treated with second-line irinotecan and Y-90 SIRT, RR was 48% with a median PFS and OS of 6 months and 12.2 months, respectively [87]. In a large, single-center, retrospective study by Saxena et al. [88], three-hundred and two patients underwent resin Y-90 SIRT for unresectable, chemo-refractory CRLM. One-hundred and forty-two patients (47%) included had two or more lines of prior chemotherapy regimens. Most patients presented with bilobar CRLM (*n* = 261, 86%), with 26 patients (9%) having >51% replacement of the liver by tumor. Over a median follow-up period of 7.2 months (range: 0.2–72.8 months), median OS was 10.5 months with a 24-month survival of 21%. On multivariate analysis, factors associated with a poorer prognosis were poor radiological response to treatment (determined on RECIST criteria), extensive replacement of hepatic parenchyma by tumor, number of previous lines of chemotherapy and low pre-procedure hemoglobin. A multi-center study by Kennedy et al. [89] evaluated 208 patients who underwent Y-90 SIRT with SIR-Spheres in patients with unresectable CRLM in a salvage setting—patients included had failed up to 3 standard lines of chemotherapy. Patients either had CT or positron emission tomography (PET) imaging follow-up within 3 months and showed a RR of 36% on CT (CR defined as disappearance of all lesions, PR defined as 50% decrease in tumor number or size by 1 measurement or necrosis of most lesions determined by Hounsfield values in the center of a lesion) and 91% RR on PET. This dramatic distinction is due to the poor sensitivity of RECIST criteria in detecting response to Y-90 SIRT, as evaluation is based on tumor size and morphology alone [98]. PET-CT has the benefit of being able to assess tumor metabolic activity, regardless of tumor size, hence increasing sensitivity to detect treatment response and has also been mentioned as a mode of imaging follow-up for Y-90 SIRT patients in the latest NCCN guidelines [13]. Overall, median survival was significantly longer in patients that responded to Y-90 SIRT, compared to non-responders (10.4 months vs. 4.5 months, *p* = 0.0001). In a multi-center study by Cosimelli et al. [90] of 50 heavily pre-treated patients who failed at least 3 lines of systemic chemotherapy (FOLFOX and FOLFIRI regimens) treated with Y-90 SIRT (majority treated with a single treatment), RR was 24% with a median PFS and OS of 3.7 months and 12.6 months, respectively. Patients who responded to SIRT on imaging also demonstrated improved OS compared to non-responders (16 months vs. 8 months, *p* < 0.0006). Other studies have also shown the safety and efficacy of Y-90 SIRT in heavily pre-treated patients besides prior systemic chemotherapy. A phase I study by Sofocleous et al. [91] evaluated 19 patients undergoing Y-90 SIRT with progression after FUDR-HAIC and systemic chemotherapy. All patients had progressed after at least 2 or more prior lines of systemic chemotherapy and more than 1 prior line of HAIC with the mean time from primary diagnosis to Y-90 SIRT being 53.8 months. Median follow-up time was 31.2 months after Y-90 SIRT, with reported median PFS and OS of 2 months and 14.9 months, respectively. Post Y-90 SIRT treatment, seventeen patients (89.5%) went on to receive further systemic chemotherapy and 9 patients (53%) went on to receive further HAIC for liver disease progression. Despite this, Y-90 SIRT did not appear to adversely impact the tolerability of future subsequent treatments. Furthermore, in more than half of the patients that received HAIC after Y-90 SIRT in this cohort, there were no cases of radiation-induced liver disease (RILD) or other significant toxicities. In a separate single-center retrospective study, Sofocleous et al. [92] evaluated the outcomes of resin Y-90 SIRT in 53 heavily pre-treated patients. In this study, besides 28% of patients having had 3 or more lines of prior systemic chemotherapy, twenty-nine patients (55%) had previous HAIC, and 26 patients (49%) had prior liver surgery. Within a median follow-up period of 15 months, median liver PFS and OS were 4.7 months and 12.7 months, respectively. Post-Y-90 SIRT, 34 patients (64%) went on to receive further additional therapy for disease progression, such as systemic chemotherapy, HAIC or thermal ablation, without any significant increase in complications or clinical toxicities. Together, these studies support the fact that Y-90 SIRT does not preclude patients from undergoing future subsequent liver-directed therapies in the event of liver disease progression. The largest series available to date evaluating the use of SIR-Spheres, the Metastatic colorectal cancer liver metastasis Outcomes after Radioembolization (MORE) study included 606 patients demonstrating a median OS of 9.6 months [93]. Y-90 SIRT was used as second-line treatment in 35.3% of patients, third-line treatment in 31.6% of patients and fourth-line treatment in 27.1% of patients. Not surprisingly, patients treated with Y-90 SIRT as second-line treatment fared better than those as fourth-line treatment (median OS of 13 months vs. 8.1 months, *p* <0.001), which was shown to be an independent predictor of improved survival. Other significant independent predictors that worsened survival were the presence of extrahepatic disease, extent of tumor burden and baseline liver function. In the only phase III RCT of 46 patients (refractory to oxaliplatin and irinotecan) by Hendlisz et al. [16], comparing Y-90 SIRT with 5-FU systemic chemotherapy compared to 5-FU chemotherapy alone [16], there was improved median PFS in the Y-90 SIRT arm compared to the chemotherapy arm (4.5 months vs. 2.1 months, *p* = 0.03), although this did not confer to a significant OS benefit (10 months vs. 7.3 months, *p* = 0.8). This may have been due to the small sample size and the lack of power to detect a significant difference [99]. Additionally, cross-over was also allowed for this study, with 10 patients (43%) in the 5-FU chemotherapy-only arm receiving further Y-90 SIRT monotherapy, which may have also confounded the OS data.

Pertaining to glass microspheres, similar results have been procured, but to a lesser degree, partly due to the different approved indications by the FDA. A study by Lewandowski et al. [94] evaluated 214 patients treated by Y-90 SIRT over a 12-year period spanning the era before the addition of biological agents: a median OS of 10.6 months was reported from the first Y-90 SIRT treatment, which was promising, considering 42% of patients had extrahepatic disease. Independent predictors of survival were similar to those seen in other large studies such as <2 prior chemotherapy lines, no prior biological agents and those who received Y-90 SIRT in the earlier stages of disease. The largest multi-center retrospective review to date evaluating the safety and efficacy of Theraspheres by Hickey et al. [95] included 531 patients and reported a similar median OS of 10.6 months from first Y-90 SIRT treatment—more than half of patients had received 3 prior systemic agents prior to Y-90 SIRT treatment (56%). Currently, a multi-center phase III RCT is underway (Efficacy Evaluation of TheraSphere Following Failed First Line Chemotherapy in Metastatic Colorectal Cancer, EPOCH trial) evaluating the efficacy of Therapshere-based Y-90 SIRT in conjunction with second-line chemotherapy vs. chemotherapy alone in patients with CRLM who have failed first-line systemic chemotherapy [102].

Several studies have specifically investigated different prognostic factors affecting the oncologic outcomes of Y-90 SIRT treatment to refine patient selection and balance the benefits of treatment response and life prolongation against the hazards of over-aggressive treatment in this cohort of chemo-refractory patients. In 2016, Damm et al. [103] analyzed 106 salvage patients (failed at least 1 line of prior systemic chemotherapy, with 28 patients, 26%, failing 4 lines or more) who underwent Y-90 SIRT. A predictive scoring system was proposed, comprising of hepatic tumor load, carcinoembryonic antigen (CEA) and/or cancer antigen 19–9 (CA 19–9), and Karnofsky Index after these factors were found to have a significant impact on OS upon multivariate Cox regression analysis. In this study, median PFS and OS were 3.5 and 6.7 months respectively, after first Y-90 SIRT treatment. This survival rate was worse when compared to other Y-90 SIRT salvage series mentioned previously, which was attributed to a potentially over-aggressive indication for Y-90 SIRT treatment. However, when applying the predictive scoring system to their own patient cohort, patients without negative predictive factors reached a median OS of 13.7 months, comparable to other salvage Y-90 SIRT series. In a more recent study, Kurilova et al. [104] sought to create a normogram including 6 preprocedural parameters to predict liver PFS and OS outcomes in chemo-refractory patients undergoing Y-90 SIRT. One-hundred and three patients with chemo-refractory CRLM who underwent Y-90 SIRT were included in this retrospective analysis. With a median follow-up time of 9.0 months, reported median liver PFS and OS were 4 and 11.3 months, respectively. Pertaining to predictive factors associated with an increased liver PFS, only baseline standard uptake value (SUVmax), a metabolic tumor marker determined on PET-CT, was found to be significant. The 6 predictive parameters significantly associated with increased OS were baseline CEA levels, baseline alanine aminotransferase (ALT) levels, albumin level, sum of sizes of the two largest CRLM diameters in the treatment region, number of extrahepatic disease sites and tumor differentiation level. After each significant factor was assigned points according to each hazard ratio, one-year OS of patients with <25 points indicated a 90% chance of 1-year survival, whereas total sum of points > 80 estimated a 10% chance of 1-year survival. Shady et al. [98] compared different metrics of metabolic response (SUVmax, SUVpeak, metabolic tumor volume (MTV) and total lesion glycolysis (TLG)) on 18-fluorodeoxyglucose (FDG)-PET imaging to determine its prognostication value in treatment response and OS. In this retrospective review, forty-nine patients with 119 target tumors were treated with resin Y-90 SIRT in a salvage setting. The different metrics were calculated at baseline and on the first follow-up PET/CT studies after Y-90 SIRT treatment to determine whether patients were either responders or non-responders to treatment. With an overall median OS of 12.7 months, univariate analysis showed that early metabolic response (when determined by MTV and TLG) significantly predicted an improved OS.

The significance of genomic mutations as independent predictors of OS in chemo-refractory CRLM patients undergoing Y-90 SIRT have also been evaluated. A retrospective study by Lahti et al. [105] evaluated the outcomes of 104 consecutive chemo-refractory CRLM patients with documented Kirsten rat sarcoma (*KRAS*) mutation status prior to undergoing resin Y-90 SIRT treatment. Most patients (84.6%) had failed at least 2 prior lines of systemic chemotherapy, with a small portion of patients having progressed despite other liver-directed therapies such as prior HAIC (16.3%) and TACE (8.7%). *KRAS* mutations were identified in 45 (43.3%) patients and median OS in mutant *KRAS* patients was approximately half that of wild-type (wt) *KRAS* patients (4.8 months vs. 9.5 months, respectively). Multivariate Cox regression analysis demonstrated mutant *KRAS* status to be an independent, negative predictor of OS in this cohort of patients, amongst other factors such as a worse Childs-Pugh score, higher baseline CEA level and no further systemic chemotherapy after Y-90 SIRT. One potential explanation for this result may be due to the intrinsic radio-resistance specific to *KRAS* mutated cell lines, which has been extensively researched in various other types of cancers where *KRAS* mutations are also prevalent. However, KRAS mutant CRC patients are also more likely to develop lung, brain and bone metastasis [106], and as such, it is unclear whether this may just be a reflection of a more aggressive biological process and advanced disease in these patients. In a similar study, Dabrowiecki et al. [107] evaluated 58 patients with known genomic analysis treated with resin Y-90 SIRT after progression of disease on at least one prior line of systemic chemotherapy. Whilst overall median OS in this study was calculated from time of diagnosis of hepatic metastasis, rather than from first Y-90 SIRT treatment, patients with CRLM without genomic mutation (mitogen-activated protein kinase (MAPK) wild-type) had prolonged OS compared to those patients with any type of mutation (median OS of 36.6 vs. 23.7 months, *p* = 0.02). Also, there was a significantly prolonged OS in patients receiving Y-90 SIRT after failing one line of prior systemic chemotherapy vs. patients receiving Y-90 SIRT after failing multiple lines of chemotherapy (median OS of 46.3 vs. 26.6 months, *p* = 0.005), thus conferring a potential survival advantage when Y-90 SIRT is administered earlier in chemo-refractory patients.

### 4.2. Evidence for Y-90 in the First-Line Setting

In 2004, van Hazel et al. [100] performed a small RCT involving 21 chemo-naïve patients with unresectable CRLM, comparing Y-90 SIRT and systemic chemotherapy (5-FU/leucovorin) with systemic chemotherapy alone. The study showed a significant improvement in RR in the Y-90 SIRT combination arm compared to chemotherapy-alone arm (PR 73% vs. 0%, *p* < 0.001), as well as improved PFS (18.6 vs. 3.6 months, *p* < 0.0005) and improved median OS (29.4 vs. 12.8 months, *p* = 0.02). Although the numbers of this study were small, the results were promising and acted as a precursor to larger phase III RCTs to investigate PFS and OS as primary endpoints.

The SIRFLOX, FOXFIRE and FOXFIRE-Global phase III RCTs were performed to evaluate the benefit of Y-90 SIRT in the first-line setting in conjunction with systemic chemotherapy (FOLFOX) in patients with unresectable CRLM [108,109,110]. The studies were performed in 14 countries, included a total of 1103 patients (randomized to either Y-90 SIRT and FOLFOX or FOLFOX alone ± biological agents) and were designed for a combined analysis of OS [101]. The pooled results showed that whilst there was significant improvement in liver disease control in the Y-90 SIRT combination arm, this did not translate to a significant improved PFS (11 vs. 10.3 months) or median OS (22.6 vs. 23.3 months). The authors thus concluded that the use of Y-90 SIRT in conjunction with systemic chemotherapy as first-line treatment in patients with liver-only or liver-dominant CRLM could not be recommended in unselected patients. Possible reasons for this outcome could be partly explained by the high proportion of patients (54%) who developed first progression at an extrahepatic site, as Y-90 SIRT would only act to treat liver disease. Also, 40% of patients in the SIRFLOX trial had limited extrahepatic disease at baseline. Further, crossover was allowed and anticipated where 12% of patients randomized to FOLFOX alone received Y-90 SIRT at a later line of therapy and 8% of patients assigned to Y-90 SIRT did not receive treatment due to unsuitability, only after randomization. Together, these factors could have also affected the primary endpoint. Others have raised concerns over the technical aspects of Y-90 SIRT used in these trials, including lack of treatment details (e.g., net administered activity, residual activity after administration, treated target volumes and lung shunts) and inadequate dose optimization [111], with the caveat that this may not have affected the large proportion of patients who developed extrahepatic progression as the first site of progressive disease. Interestingly, in a separate analysis of the SIRFLOX and FOXFIRE-Global trials, Gibbs et al. [112] showed that patients with right-side primary (RSP) tumors benefited from Y-90 SIRT, demonstrating a significantly improved OS of 4.9 months (median, 22 vs. 17.1 months, Y-90 SIRT combination arm vs. chemotherapy alone, *p* = 0.008) when compared to patients with a left-side primary (LSP) tumor (24.6 vs. 26.6 months, *p* = 0.264). This consistent gain in OS was demonstrated in both trials (SIRFLOX, 4.8 months gain and FOXFIRE-GLOBAL, 7.9 months gain), suggesting that this result was not related to a consequence of a change imbalance of prognostic factors. Furthermore, multivariate analysis into factors impacting OS in RSP patients showed that treatment with Y-90 SIRT remained statistically significant. Whilst the authors concluded that there was a lack of a full biological understanding for this side-dependent difference, one potential explanation may be that more aggressive, initial systemic therapies (e.g., triplet chemotherapy regimen with bevacizumab) used in the first-line setting could be having a greater impact in this cohort of patients who already have a known poorer prognosis [113,114] when compared to patients with LSP tumors. However, given that many patients may not be fit for a triplet chemotherapy regimen and given the lack of survival benefit with EGFR inhibitors in certain patients with RSP tumors (*RAS* wild-type tumors) [114], there may be a potential role for the inclusion of Y-90 SIRT as part of first-line treatment in certain patients with RSP tumors with liver-only or liver-dominant metastatic disease. Further studies evaluating the impact of Y-90 SIRT on primary tumor side, as well as other potential drivers of side-based differences in patients with metastatic CRC, is warranted.

An important secondary objective in the clinical trials was to determine the impact of Y-90 SIRT and FOLFOX on QoL compared to patients on FOLFOX alone. The results of a QoL study on the combined trials [115] demonstrated an initial, significantly worse QoL in the combination Y-90 SIRT and FOLFOX arm when compared to the FOLFOX arm alone, up to 3 months after Y-90 SIRT administration, although these changes did not reach thresholds for clinical importance. Whilst patients in the Y-90 SIRT combination arm experienced more fatigue, which is a common side effect of Y-90 SIRT, this cohort of patients experienced significantly lower levels of other side effects seen in the FOLFOX alone group (e.g., sore mouth/tongue and peripheral neuropathy), likely due to a dose reduction of oxaliplatin mandated for three cycles for patients in the Y-90 SIRT combination arm. After the initial 3-month period after Y-90 SIRT, there were no clinically significant differences in QoL between the two study arms. Overall, the study concluded that there was no significant detriment in QoL after the initial 3 months post-Y-90 SIRT that were deemed clinically significant.

### 4.3. Y-90 SIRT in The Neoadjuvant Setting (Radiation Lobectomy and Radiation Segmentectomy) 

In patients with initially unresectable CRLM, shrinkage of tumorous tissue and disengaging it from vital structures can increase the chance of the patient being converted to hepatic resection [116]. Another critical factor is for patients to have an adequate future liver remnant (FLR) volume to avoid liver failure and death post-hepatectomy. When an adequate FLR is lacking, several preoperative measures can be performed to improve liver reserves and contralateral hypertrophy to allow for safe hepatic resection [117]. These include Y-90 SIRT, portal vein embolization (PVE) and associating liver partition with portal vein ligation (PVL) for staged hepatectomy (ALPPS) [118,119,120]. Y-90 SIRT, when used in this setting, is also termed radiation lobectomy (RL), which was first described by Siddiqi et al. in 2009 as a palliative treatment for CLRM [121]. In distinction to conventional Y-90 SIRT treatment, RL delivers an intentionally higher ‘ablative’ radiation dose (at least 120 Gy) to both tumor and adjacent liver parenchyma. The benefit of this is two-fold; firstly, this has the benefit of being able to offer local tumor control to the treated side, while simultaneously permitting contralateral lobe FLR hypertrophy, and secondly, as the FLR hypertrophy usually occurs over several months after RL, this allows a biological test of time to assess disease progression, thus preventing morbidity from unnecessary hepatectomy. If the FLR hypertrophy is insufficient after RL, this also does not preclude the patient from undergoing future subsequent procedures to induce further contralateral hypertrophy. In a recent systematic review [122] of 16 studies comprising 602 patients, evaluating contralateral liver hypertrophy and oncological outcomes following RL, the median kinetic growth rate per week of the contralateral liver lobe was 0.7%, with the maximum degree of contralateral hypertrophy exceeding 40% achieved after 9 months. Further, reported local tumor control was 84%, with CTR achieved in 30% of patients. Moreover, a recent secondary analysis [123] of the SIRFLOX study patient cohort revealed a higher resectability rate in the Y-90 SIRT and FOLFOX arm compared to the FOLFOX arm alone (38.1% vs. 28.9%, *p* < 0.001), lamenting its downstaging potential.

In patients with limited unresectable CRLM, aggressive local disease control via ablative techniques in addition to systemic chemotherapy have shown a PFS and OS benefit when compared to patients treated with systemic chemotherapy alone, as demonstrated in the CLOCC (Chemotherapy and Bevacizumab With or Without Radiofrequency Ablation in Treating Unresectable Liver Metastases in Patients With Colorectal Cancer) trial [124]. In this regard, radiation segmentectomy (RS) is a potentially useful technique in patients with tumors localized to 1 or 2 segments of the liver, that are unsuitable for ablation or resection, either due to large tumor size or an unfavorable anatomical location. The concept of RS involves delivering a high ‘ablative’ dose of radiation to the involved tumor segments, resulting in tumor eradication while confining the radiation effects only to the segments infused [85,125]. Via this technique, median tumor dose can reach up to 1200 Gy, resulting in a high degree of histological necrosis and objective RR of up to 86% (according to WHO and EASL [European Association for the Study of the Liver] criteria) [125,126]. While most data published on RS is mainly in the context of HCC, several small studies have shown promising results in the context of CRLM. In a small, single-center study of 10 patients by Meiers et al. [127], RS was performed for 10 patients with hepatic metastasis (seven of which were CRLM) deemed unfit or ablation or surgery. With a mean tumor dose of 261 Gy, five out of ten patients showed a complete metabolic response according to PET response criteria in solid tumors (PERCIST), with a mean PFS of 7.1 months. In a separate study by Kurilova et al. [128], 10 patients with 14 CRLM underwent RS, demonstrating a response rate of 100% and 44% according to the per Choi and RECIST 1.1 criteria, respectively. With a median deliver dose of 293 Gy, reported median OS in this small cohort was 41.5 months, with a 2-year local tumor control rate of 83%. In short, the concept of RS in the context of CRLM is still in its infancy, although initial results on safety and efficacy are encouraging and it serves a potential role in patients with limited liver metastatic disease unamenable to surgery or local ablative techniques. 

### 4.4. Complications of Y-90 SIRT

In general, Y-90 SIRT is safe and well-tolerated, although there are specific complications related to this therapy worth mentioning. Similar to TACE, post-Y-90 SIRT patients can develop a post-radioembolization syndrome (incidence 20–70%), which can manifest as fatigue, nausea, vomiting, transient liver dysfunction, abdominal discomfort, cachexia and fever up to 2 weeks after the procedure [129]. This is akin to post-embolization syndrome commonly seen in post-TACE patients, although usually milder in severity and usually does not require hospitalization [89]. The effects of aberrant radioactive microspheres depositing elsewhere besides the liver can result in gastrointestinal ulceration (<5%) which can be severe, cholecystitis (<1%), pancreatitis (<1%) and radiation dermatitis (usually via the falciform artery supplying the anterior abdominal wall, <1%). Biliary complications can occur in up to 10% of patients, and whilst the majority of patients may be asymptomatic, this can present as cholangitis, stricture formation or biloma secondary to biliary necrosis. The rate of biliary complications is also significantly higher in patients with a hepaticojejunostomy or prior instrumentation to the ampulla of Vater [130]. Radiation pneumonitis has been described in patients with a high LSF (>13%) developing restrictive ventilatory dysfunction post-Y-90 SIRT, although the incidence of this is rare (<1%) using standard dosimetry models [130]. One of the most serious complications related to Y-90 SIRT is radiation-induced liver disease (RILD), due to excessive radiation dose to the liver parenchyma. This is reported in 0–4% of patients in the literature and in the most severe cases, can manifest as fulminant liver failure with jaundice and ascites, 4–8 weeks after treatment [130]. Factors associated with RILD are patients with pre-existing liver cirrhosis, baseline bilirubin levels more than 2 mg/dL, single-session whole liver radioembolization and inaccurate dosimetry calculations.

## 5. Conclusions

The management of CRLM remains a major challenge despite the constantly evolving landscape of improving loco-regional and systemic therapies. Currently, in appropriate patients with chemo-refractory disease with liver-only or liver-dominant metastatic disease, guidelines recommend considering the use of hepatic-directed IATs [13,14]. In chemo-refractory patients, systemic treatment options are limited, providing a median OS of 4–6 months when compared to best supportive care [96,97]. As such, in this select cohort of patients, it would seem reasonable to consider liver-directed IATs to provide local disease control and to prolong OS. However, most available data in the literature is based either on retrospective series or small, prospective phase I/II trials, with few large-scale RCTs available in the literature, which are desperately needed to increase the robustness of the data to support these therapies in this setting. Moreover, unlike systemic chemotherapeutic regimens which have a broad treatment algorithm, there is currently little consensus on the optimal role, choice of IAT and timing for use within this cohort of patients. To date, there have been two systemic reviews which have tried to address this issue [131,132], although in both instances, due to significant heterogeneity encountered in the available literature, this precluded a meaningful comparison between the different IATs and evidence supporting one over the other, and optimal timing for use is still lacking. Two small retrospective studies have also compared the effectiveness of HAIC vs. Y-90 SIRT in unresectable, pre-treated CRLM [133,134]. Despite one of the studies [133] suggesting HAIC to be associated with a better OS when compared to Y-90 SIRT, it is noteworthy that this is most likely related to lead time bias, as when examining the two groups, it is apparent that Y-90 SIRT was likely offered at a later time during the natural history of the disease when compared to HAIC. Further, the difference in OS is only apparent upon diagnosis of ‘isolated, unresectable CRLM’, a rather random time point of assessment, with no significant OS difference from time of stage 4 diagnosis between the HAIC and Y-90 SIRT group. In short, it is difficult to draw any significant conclusions from this particular study. It is thus likely that the current choice of IAT would highly depend on the technical expertise available, as well as cost considerations in certain countries. There has also been increasing enthusiasm in expanding the role of IATs earlier in the course of disease as an addition to systemic chemotherapy to intensity treatment response, either in the first-line setting in conjunction with systemic treatment, or with the aim of potential downstaging for surgical resection. In recent years, the largest study in the field of interventional oncology pertaining to the use of Y-90 SIRT in the first-line setting in conjunction with systemic chemotherapy [101] wielded disappointing results with an absence of OS benefit, and as such, was not recommended in unselected patients with metastatic CRC. In this regard, it is likely that Y-90 SIRT would still provide the most benefit in the treatment of chemo-refractory patients. Future directions in this field would include large-scale, multi-center RCTs comparing IATs with concurrent systemic chemotherapy to systemic chemotherapy alone, as well as direct comparison between IATs with a strict patient selection criterion and specified clinical outcomes. Finally, future research would also include the investigation on the impact of genetic determinants on treatment response with the aim of identifying optimal treatment populations that would benefit most from these therapies.

## Figures and Tables

**Table 1:** Selected prospective trials and retrospective studies for Hepatic Arterial Infusion Chemotherapy (HAIC) with systemic chemotherapy.

Author and Year Published	Study Design	No. of Patients	Study Arm	Prior Lines of Systemic Chemotherapy, No. of Prior Lines: No. of Patients				RR, %	PFS, mo	OS, mo	Conversion to Resection, %
				None	1	2	3 or More				
Kemeny 2005 [28]	Prospective, P-I	36	Arm 1: HAI FUDR + SYS OXA/IRI Arm 2: HAI FUDR + SYS OXA/FU/LV	4	32	-	-	90 86	16.4 9.4	35.8 22	19 ^a^
Kemeny 2009 [29]	Prospective, P-I	49	HAI FUDR/Dex + SYS OXA/IRI	23	26			92	NR	39.8	47 ^b^
Goere 2010 [30]	Prospective, P-I	87	HAI OXA + SYS 5-FU/LV	18	69			55	NR	NR ^c^	26 ^d^
Lévi 2011 [31]	Retrospective	56	Chronomodulated HAI combination of 5-FU/IRI/OXA + SYS CET	-	8	12	36	32.1	4.6	13.7	11
Lévi 2016 30 [32]	Prospective, P-II	64	HAI 5-FU/IRI/OXA + SYS CET	-	28	36		41	9.3	25.7	30
Cercek 2016 [33]	Retrospective	110	HAI FUDR + BEST SYS chemotherapy ^e^				110 ^f^	35	5	16.3	NR
Pak 2018 [34]	Prospective, P-II	64	HAI FUDR + Best SYS chemotherapy ^g^	21	30	12	1	73	13	38	52

Abbreviations: HAI; Hepatic arterial infusion; FUDR, Flurodeoxyuridine; Dex: Dexamethasone; SYS, systemic; IRI, Irinotecan; OXA, Oxaliplatin; LV, Leucovorin; 5-FU, 5-Flurouracil; CET, Cetuximab; RR, Response rate; PFS, Progression free survival; OS, Overall survival; NR, not reported, ^a^ 7/21 patients in Arm 1 down-staged to resection, ^b^ CTR was higher in the chemo-naïve group, 57%, ^c^ 5-year OS: 56%, ^d^ CTR was higher in the chemo-naïve group, 53%, ^e^ Either 5-FU/ LV, FOLFOX, FOLFIRI, IRI or Capectabine based on prior chemotherapy history ± BEV and anti-EGFR agent (if *RAS* status was known), ^f^ All patients progressed on at least 3 lines of prior systemic chemotherapy (5-FU, OXA, IRI), ^g^ Either OXA/IRI or 5-FU/ LV/IRI based on prior chemotherapy history ± BEV.

**Table 2:** Selected prospective trials for DEBIRI TACE.

**Author and Year Published**	**Study Design**	**No. of Patients/No. of Treatments**	**Study Arm**	**Liver Metastatic Involvement,** **No. of Patients**			**Prior Lines of Systemic Chemotherapy,** **No. of Prior Lines: No. of Patients**				**RR, %**	**PFS, mo**	**OS, mo**	**AE** **G 1–2**	**SAE** **G 3–4 or Above**
				**<25%**	**26–50%**	**>50%**	**None**	**1**	**2**	**3 or More**					
Martins 2011 [65]	Propsective, P-I	55/99	DEBIRI-TACE	30	13	12	-	17	38	-	75 (1 year)	11	19	28%	7%
Aliberti 2011 [66]	Prospective, P-II	82/185	DEBIRI-TACE	Median 33% (range 25–50%)			-	-	82		78 (3 mo)	8	25	NR	25% ^a^
Fiorentini 2020 [68]	Propspective, Observational RCT	30	Arm 1: DEBIRI-TACE Arm 2: DEBIRI-TACE + BEV	Range 25–40%				4	11	15	19 77	4 6	5.8 12	12% 16%	0%
Fiorentini 2012 [69]	Phase III RCT	74	Arm 1: DEBIRI-TACE Arm 2: SYS FOLFIRI	26 26	10 12	0 0	-	-	36 38	-	68.6 20	7 4	22 15	14% 18%	2% 4%
**Evidence for DEBIRI-TACE in conjunction with systemic chemotherapy in the first-line setting**															
**Author and Year Published**	**Study Design**	**No. of Patients/No. of Treatments**	**Study Arm**	**Liver Metastatic Involvement,** **No. of Patients**			**RR, %**			**PFS, mo**	**OS, mo**		**AE** **G 1–2**	**SAE** **G 3–4 or Above**	**CTR, %**
				**<25%**	**26–50%**	**>50%**									
Martins 2015 [70]	Phase II RCT	70	Arm 1: DEBIRI-TACE + mFOLFOX ± BEV Arm 2: SYS FOLFOX ± BEV	Median involvement, 30% in both arms			76 60			15 ^b^ 8	NR		973 events 459 events	57 events 15 events	35 16
Pernot 2020 [71]	Prospective P-II	57	DEBIRI-TACE + mFOLFOX6	Bilobar disease in 88% of patients			73			10.8	37.4		75.4%	67% ^c^	33

Abbreviations: DEBIRI, drug-eluting beads, Irinotecan; TACE, trans-arterial chemoembolization; SYS, systemic; BEV, Bevacizumab; mo, month; AE, adverse effect; SAE, severe adverse effect (Based on Common Terminology Criteria for Adverse Events (CTCAE v5.0); NR, not reported, ^a^ 25% reported as grade 3, right upper quadrant pain; no G 4–5 events reported, ^b^ Result was not statistically significant (15 vs. 12 months, *p* = 0.18), ^c^ More significant grade 3–5 SAE were seen when DEBIRI was performed for a bilobar approach vs. unilobar approach (87.5% vs. 47.2%).

**Table 3:** Selected prospective trials and retrospective studies for Y-90 SIRT.

**Author and Year Published**	**Study Design**	**No. of Patients**	**Study Arm**	**Liver Metastatic Involvement,** **No. of Patients**			**Prior Lines of Systemic Chemotherapy/Patients**				**Mean Activity (Gbq)**	**RR, %**	**PFS, mo**	**Liver PFS, mo**		**OS, mo**		**AE** **G 1–2**	**SAE** **G 3–4 Above**
				**<25%**	**26–50%**	**>50%**	**None**	**1**	**2**	**3 or More**									
Gray 2001 [15]	Phase III RCT	74	Arm 1: Y-90 SIRT (Resin) + HAI FUDR Arm 2: HAI FUDR	NR	NR	NR	-	5 5			2.2	37 14	NR	15.9 9.7		17 15.9 *		NR	64% 66%
Kennedy 2005 [89]	Prospective, P I-II	208	Y-90 SIRT (Resin)	NR	NR	NR	-	-	198	10	1.75	36	NR	NR		10.5 (R) 4.5 (NOR)		24.5%	28.5%
Cosimelli 2010 [90]	Prospective	50	Y-90 SIRT (Resin)	20	30	0	-	-	-	50	1.7	24	3.7	NR		12.6		42%	4% ^a^
Kennedy 2015 [93]	Retrospective	606	Y-90 SIRT (Resin)	388	148	22	35	206	184	158 ^b^	1.46	NR	NR	NR		9.6		41.4% ^c^	10.2% ^d^
Hendlisz 2010 [16]	Phase III RCT	44	Arm 1: Y-90 SIRT (Resin) + SYS 5-FU Arm 2: SYS 5-FU	NR	NR	NR	-	20 13			1.79	10 0	4.5 2.1	5.5 2.1		10.0 7.3 *		NR	5% 27%
Lewandowski 2014 [94]	Prospective	214	Y-90 SIRT (Glass)	174	31	9	-	14	35	160	2.35	NR	NR	NR		10.6		50%	39% ^e^
Hickey 2016 [95]	Retrospective	531	Y-90 SIRT (Glass)	370	103	58	15	216		295	NR	NR	NR	NR		10.6		55%	13%
**Evidence for Y-90 SIRT in conjunction with systemic chemotherapy in the first-line setting**																			
**Author and Year Published**	**Study Design**	**No. of Patients**	**Study Arm**	**Liver Metastatic Involvement,** **No. of Patients**			**Mean Activity (Gbq)**			**RR, %**	**PFS, mo**		**Liver PFS, Mo**		**OS, mo**		**AE** **G 1–2**	**SAE** **G 3–4 or Above**	
				**<25%**	**26–50%**	**>50%**													
van Hazel 2004 [100]	Phase II RCT	21	Arm 1: Y-90 SIRT (Resin) + SYS 5-FU/ LV Arm 2: SYS 5-FU/LV	8 7	3 3		2.25			73 0	11.5 4.6		NR		29.4 12.8		5 events	13 events	
Wasan 2017 [101]	Phase III RCT	1103	Arm 1: Y-90 SIRT (Resin) + SYS FOLFOX Arm 2: SYS FOLFOX	374 380	374 179		NR ^f^			72 63	11 10.3 *		20.5 12.6		23.4 23.9 *		26% 33%	74% 67%	

Abbreviations: SIRT, Selective internal radiation therapy; RCT, randomized control trial; 5-FU, Flurouracil; LV, leuvocorin; Gbq, Gigabecquerel; Gy, Gray; R, responder; NOR, non-responder; mo, month; AE, adverse effect; SAE, severe adverse effect (Based on Common Terminology Criteria for Adverse Events (CTCAE v5.0); NR, not reported, * Results not statistically significant, ^a^ 2 patients died: The first patient died after 40 days from kidney failure, the second patient died after 60 days from liver failure, both were classified as possibly related to treatment, ^b^ 23 patients did not have data on prior systemic chemotherapy treatment, ^c^ Most common G1-2 AE was related to gastrointestinal symptoms followed by constitutional symptoms, including fatigue in 39.8% of patients, ^d^ Most common G3 or above AE was related to gastrointestinal symptoms followed by hepatobiliary-related AE in 8.6% of patients, ^e^ Most common was G3 absolute lymphocyte, bilirubin, albumin, alkaline phosphatase (ALP) and aspartate aminotransferase (AST) toxicities. Grade 4 absolute lymphocyte and ALP toxicities observed in 5%., ^f^ Median 1.4 GBq (range 0.4–3.1 Gbq) reported in the SIRFLOX study only.
